# Frequent MGMT (0^6^-methylguanine-DNA methyltransferase) hypermethylation in long-term survivors of glioblastoma: a single institution experience

**DOI:** 10.2478/v10019-010-0023-y

**Published:** 2010-05-24

**Authors:** Martina Baur, Matthias Preusser, Maria Piribauer, Katarzyna Elandt, Marco Hassler, Marcus Hudec, Christian Dittrich, Christine Marosi

**Affiliations:** 1 Applied Cancer Research-Institution for Translational Research Vienna (ACR-ITR VIEnna/CEADDP), Vienna, Austria; 2 Ludwig Boltzmann Institute for Applied Cancer Research (LBI-ACR VIEnna)-Ludwig Boltzmann Cluster Translational Oncology, Kaiser Franz Josef-Spital, Vienna, Austria; 3 Department of Internal Medicine I, Medical University Vienna, Vienna, Austria; 4 Institute of Neurology, Medical University Vienna, Vienna, Austria; 5 Department of Scientific Computing, University of Vienna, Vienna, Austria

**Keywords:** glioblastoma multiforme (GBM), high grade glioma, MGMT promoter methylation, hypermethylation, long-term survival

## Abstract

**Background:**

The aim of this retrospective study was to analyse the MGMT (0^6^-methylguanine-DNA methyltransferase) promoter methylation status in long-term surviving (≥ 3 years) patients with glioblastoma multiforme (GBM).

**Methods:**

The methylation status of the MGMT promoter was determined by bisulfite modification of the DNA and subsequent methylation-specific polymerase-chain-reaction (MSP). DNA was extracted from routinely formalin-fixed and paraffin-embedded tumour tissue samples.

**Results:**

MSP yielded interpretable results in only 14 of 33 (42%) long-term surviving patients with GBM. A methylated band was seen in 3 of 14, methylated as well as unmethylated bands in 8 of 14 and an only unmethylated band in 3 of 14 patients, thus, yielding MGMT promoter methylation in 11 of 14 patients. The two groups of patients with methylated and unmethylated MGMT promoter status were too small to draw any firm statistical conclusions.

**Conclusions:**

Long-term surviving patients with GBM have very frequently intratumoural MGMT promoter methylation. This phenomenon discriminates long-term survivors from a non-selected group of patients with GBM. The standardization of the MSP for the determination of the MGMT promoter methylation status seems to be necessary in order to make this methodology a more reliable one.

## Introduction

Glioblastoma multiforme (GBM) is the most common primary brain tumour in adults. It represents the most frequently encountered type of glial tumours and can also occur in children.^1^,^2^ Median survival is generally only slightly longer than one year based on multimodal approaches consisting of maximal feasible resection, radiotherapy and chemotherapy. A substantial step forward in the treatment of GBM was reached by the randomized phase III trial by Stupp *et al.,* demonstrating a significantly longer survival in patients treated with temozolomide in addition to radiotherapy followed by adjuvant temozolomide with a median survival of 15 months and a five-year survival rate of 9.8%.^3^

Distinct from unselected GBM patients, who survive about one year, there is a small subgroup of 1% – 5% of patients with GBM that survive at least 3 years after the diagnosis of GBM.^4^–^12^ They are designated as long-term glioblastoma survivors. This period of 36 months survival was also adopted in our study as the lower limit for long-term surviving GBM patients; yet, there is no generally accepted definition. All histologic diagnoses of the putative long-term surviving GBM patients have to be reviewed because in about one half of the cases the histologic diagnosis of GBM is reclassified to represent a less malignant tumour, namely oligodendroglioma, malignant mixed oligodendroglioma-astrocytoma or anaplastic astrocytoma.^6^–^10^,^13^ Although the histologic aspect of the tumours from long-term survivors does not differ from that of classical survivors, it is postulated that the long-term surviving patients are a subgroup of GBM patients with a different biological behaviour, a different therapeutic responsiveness and a distinct genetic characterization.

Clinical parameters such as young age, high Karnofsky performance status and the extent of radicality of surgery are associated with a better prognosis despite the histology of a GBM.^4^,^6^–^8^,^10^,^14^,^15^ Scott *et al.* found additional factors as the neurologic function and the dose of radiotherapy applied in their recursive partitioning analysis to be important prognostic variables.^16^ The period of symptoms before the diagnosis in long-term GBM survivors in contrast to average GBM patients is significantly longer.^10^ A significantly lower Ki-67-labeling index compared to controls has been described in tumours from long-term survivors.^10^ Such patients exhibit fewer genetic aberrations than typical GBM patients.^7^ Like in oligodenroglioma patients the loss of 19q is exclusive to the long-term survivors.^1^ Usually 6q loss, 10q loss and 19 q gain are associated with the short-term survival^7^ whereas mdm2 overexpression is less likely exhibited by the long-term GBM survivors.^5^ Molecular parameters, which can determinate the step of tumour malignancy^17^, are also important in GBM patients.^5^ The overexpression of the protein p53 and the nuclear p53 expression are significantly more frequently found in long-term surviving patients.^5^ A better molecular characterization of long-term GBM patients is achieved by examining of multiple markers suggesting that differing patterns of genetic lesions may discriminate between the long and the short-term survival of GBM patients.^7^

It has become clear that cancers in general arise from both genetic and epigenetic changes. Epigenetic changes, such as hypermethylation, may inactivate genes without changing the base sequence. Analysing a different promoter methylation status of key regulator genes implicated in apoptosis and inflammation hypermethylation of TMS1/ASC was significantly more frequent in long-term surviving GBM patients and DAPK promoter hypermethylation was only found in the long-term subset compared to unselected GBM patients.^4^ Martinez *et al.*^18^ found a significantly higher methylation rate of MGMT in long-term GBM patients compared to unselected GBM patients. The MGMT gene is located on chromosome 10q26. Methylation of the gene promoter is associated with the loss of MGMT expression which results in diminished DNA-repair activity. Tumour cells lacking MGMT are prone to cell death induced by alkylating substances such as temozolomide. In this process the alkyl-group is transferred to the active site of the MGMT protein that thereby becomes irreversibly inactivated and subsequently degraded, requiring resynthesis. Although O^6^-methylguanine accounts for less than 10% of the lesions induced by alkylating agents, it plays a major role as a trigger for cytotoxicity and apoptosis. If left unrepaired, e.g. due to epigenetic silencing of the MGMT gene or depletion the MGMT protein by saturation of the process, O^6^-methyl guanine persists in the DNA.^19^ Recently, Hegi *et al.*^20^, Glas *et al.*^21^ and Sonoda *et al.*^22^ described promoter methylation of MGMT as an independent favourable prognostic factor. Patients with GBM containing a methylated MGMT promoter benefited from temozolomide, whereas those who did not have a methylated MGMT promoter did not have such a benefit.^20^

To further characterize long-term glioblastoma patients genetically we investigate retrospectively the MGMT promoter methylation status by the bisulfite modification of the DNA and subsequent methylation-specific polymerase-chain-reaction (MSP) in formalin-fixed and paraffin-embedded tumour tissue samples of 33 long-term survivors with GBM from a single centre.

## Patients and methods

### Patient recruitment

Primary and secondary GBM patients surviving longer than 36 months after the diagnosis were retrospectively identified in a single centre, the Department of Internal Medicine I, University of Vienna, Vienna, Austria starting from the year 1995 up to 2003. The histologic diagnosis of GBM according to the World Health Organization (WHO) classification of the brain tumours was confirmed by the pathology review by M.P. All patients have been treated with alkylating agents. The clinical data were evaluated by checking patients` records, the presence and the extent of oedema by reviewing the radiologic films. A cognitive impairment was assessed by analysing the dialogues between the treating physicians and the patients; additionally, the functional capacities regarding ADL (activities of daily living) and IADL (instrumental activities of daily living) documented as reported by the relatives were scored. This study has been approved by the local ethics committee and has been performed in accordance with the ethical standards laid down in the 1964 Declaration of Helsinki. The informed consent for samples and the data analysis from each patient had been obtained.

### MGMT promoter methylation analysis

A MGMT promoter methylation status was analysed using methylation-specific PCR (MSP) as described by Hegi *et al.*^20^ In brief, genomic DNA was isolated from paraffin sections of GBM tissue using Ex-Wax DNA Extraction (Chemicon, Temecula, California, USA). The DNA was subjected to bisulfite treatment at 56˚C for 16–20h. Then the DNA was purified using Wizard DNA Clean-Up System A7280 (Promega, Madison, Wisconsin, USA). MSP was performed in a two-step “nested” approach using previously defined primer sets.^20^ The PCR products were separated on two percent agarose gels. A glioblastoma case with a known methylated MGMT promoter was used as the positive control and water was used as the negative control for MSP analysis.

### MIB-1 proliferation index immunohistochemistry

Tumour sections (3–5 micrometers thick) were immunostained with a monoclonal mouse anti-Ki-67 antibody (clone MIB-1, Dako, Glostrup, Denmark) at a dilution of 1:50 for 25 minutes. For the determination of the MIB-1 proliferation index, the fraction of labelled nuclei per 500 tumour cell nuclei was manually counted using an eye grid and was expressed as percentage.

### Statistical analysis

Time to progression reached from the date of the first neurosurgical procedure or diagnosis of glioblastoma to the time of the first objective evidence of tumour progression or the time of censoring. Survival time was defined as the time lapse from the initial surgery or diagnosis to the patient’s death or the time of censoring. Time to progression and survival were estimated using the Kaplan-Meier method. The influence on time to progression and overall survival by sex, age, presence of primary or secondary glioblastoma, side and region of the brain of the primary tumour, presence of cognitive impairment, presence of oedema and Karnofsky performance status was calculated by the log-rank test. For the analysis of the influence of the age of the formalin-fixed or paraffin embedded tumour tissue on the feasibility of determination of the MGMT promoter methylation status a χ^2^-test was used. All statistical analyses were performed using the SPSS software 15.0.

## Results

In this retrospective analysis 35 long-term surviving patients with GBM were identified from one centre. 33 of them were confirmed GBM patients after the histologic review indicating a percentage of 6% of revised histologies. The two diagnoses of the reclassified histologies were anaplastic oligodendroglioma and anaplastic astrocytoma. As 40 patients per year with primary GBM are treated in the institution this results in about four patients per year becoming long-term surviving GBM patients. This corresponds to an estimated percentage of 10% of long-term survivors in the institution. The median follow-up was 54.2 ±SD 26.1 months. The patient’s characteristics are shown in Table 1.

### Karnofsky performance score

The postoperative Karnofsky performance score of the long-term surviving patients was at least 80%. At the time of writing two women and three men were professionally still active, the two women as computer clerks with full time employment, one of the men as a teacher for mathematics in a vocational school, one as a farmer and the third as a pizza cook.

### Local relapses

20 of 33(61%) patients suffered a local relapse, eleven of them after gross total resection of the primary tumour.

### Median time to progression

**FIGURE 1:**
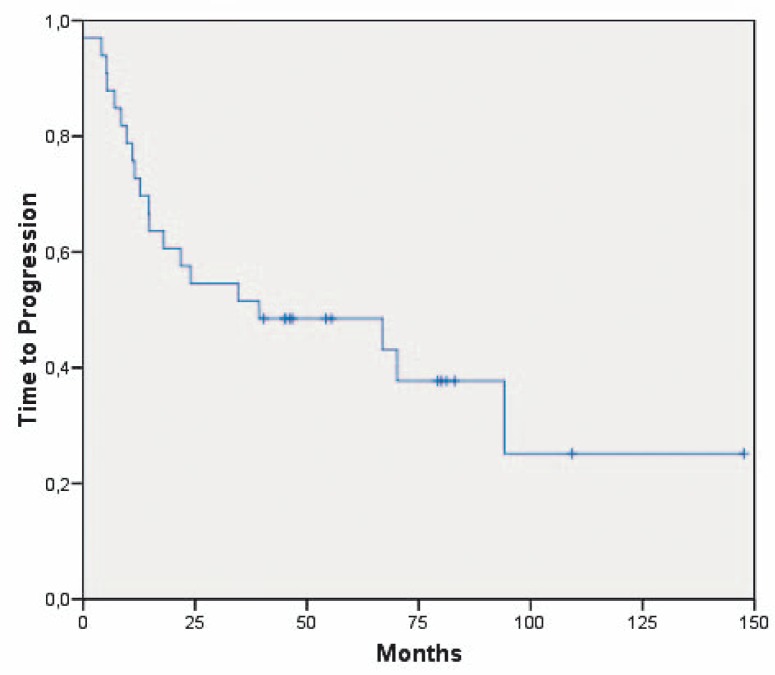
Time to progression of all long-term surviving patients with glioblastoma multiforme (n=33).

The median time to progression (TTP) was 39 months [95% CI: 0; 105.6] (Figure 1). Patients with a biopsy at initial diagnosis had a median TTP of 5.3 months [95% CI: 0; 17.8 months] (n=4), patients with a subtotal resection had a TTP of 39.3 months [95% CI: 0–92 months] (n=10) and patients with a total resection one of 66.9 months [95% CI: 0; 145.3 months] (n=19), respectively. The clinical parameters age, sex, oedema, side and region of the brain of the primary tumour and Karnofsky performance status did not impact on the TTP. Patients with the unmethylated MGMT promoter had a time to progression of 7, 39 and 79+ months whereas patients with methylated MGMT promoter had a TTP of 5 to 56+ months.

### Survival

**FIGURE 2:**
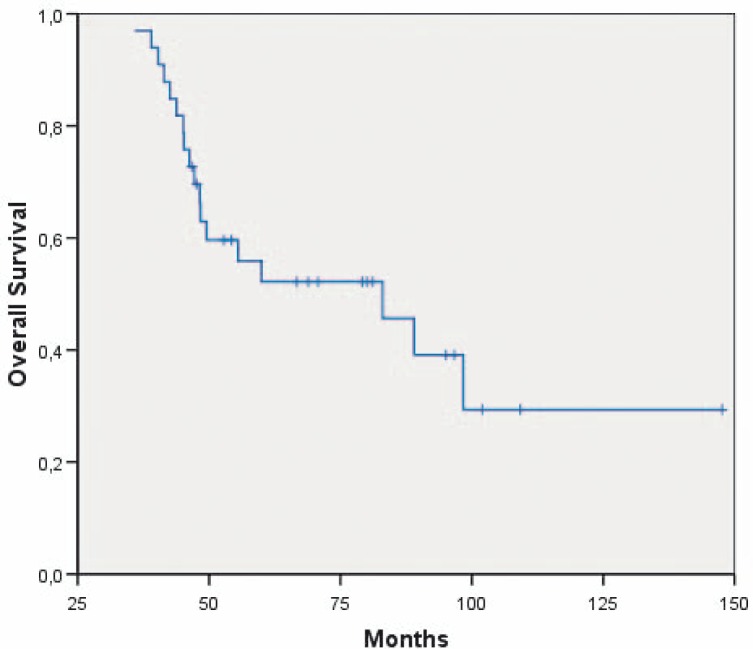
Survival of all long-term surviving patients with glioblastoma multiforme (n=33).

At the time of evaluation, 15 patients were alive, seven of them without tumour recurrence for up to 151+ months. The median survival was 83 months [95% CI; 43.8–122.3] (Figure 2). Patients with a subtotal resection survived 47.2 months [95% CI: 28.8–65.6 months] and patients with a total resection 83.0 months [95% CI: 43.2; 122.9 months], respectively. The clinical parameters age, sex, oedema, side and region of the brain of the primary tumour and Karnofsky performance status did not impact on the survival. Of note, nine of the patients with a local relapse survived longer than five years. Patients with the unmethylated MGMT promoter had a survival of 43, 79+ and 97+ months, respectively. Patients with the methylated MGMT promoter had a median survival of 48 ± SD 0.97 months.

### MIB-1 scoring

In seven patients the immunohistochemical staining of MIB-1 was determined. The mean MIB-1 score was 29.1%, the median 30.3% (range 12.1 – 49%).

### MGMT promoter methylation status (Table 2)

Only in 14 of 33 (42%) patients the determination of the MGMT promoter methylation status by MSP yielded interpretable results. There was no linear correlation of the success rate to the age of the paraffin block (p=0.5). Of 14 patients with interpretable MSP results, three of 14 patients had a methylated MGMT promoter, three of 14 patients an unmethylated MGMT promoter and 8 of 14 patients partly a methylated and partly an unmethylated MGMT promoter. Thus, the MGMT promoter methylation was found in 11 of the 14 patients. These two groups of patients with the methylated and the unmethylated MGMT promoter status, respectively were too small to draw reliable conclusions based on statistical testing.

## Discussion

Reports on the MGMT promoter methylation status in long-term surviving patients with glioblastoma multiforme are scarce. 78.5% of long-term survivors presented with MGMT promoter hypermethylation. This is in the same range as reported by Martinez *et al.,* Sonoda *et al.* and Krex *et al.*^18^,^22^.^23^ This high proportion of patients with the MGMT promoter methylation is in clear contrast the 44% (range 25– 68%) determined from 13 different studies of unselected patients with GBM.^18^,^20^,^24^,^34^ However, the high rate of methylated tumours in the long-term surviving patients let suggest that MGMT promoter methylation is of paramount importance for response to the actual standard therapy with alkylating agents in GBM.^3^ The proof of this principle is eagerly awaited in form of the results of the prospective Intergroup trial RTOG0525/EORTC26052 which will not yet be presented at ASCO 2010 testing dose-intense temozolomide in comparison to standard-dose temozolomide dependent on the MGMT promoter methylation status in GBM patients.

Although 33 patients were initially included in our analysis of long-term surviving patients with GBM, the paraffin embedded tissue blocks of only 14 out of 33 (42%) patients were suitable for the MGMT hypermethylation test by MSP. Because of the small number of patients it was impossible to determine whether the MGMT methylation status was of prognostic impact in our patient cohort. The MGMT promoter methylation status as a prognostic factor in long-term surviving GBM patients should be further evaluated in prospective studies.

The statistical analysis did not show a significant difference between older paraffin embedded tissue in contrast to younger patient samples (p=0.5); however, only higher patient numbers in the subgroups could provide reliable significant results. The success rate of the methylation specific PCR determination on paraffin-embedded tumour samples is highly variable and centre dependent.^20^ Hegi *et al.* reported on a median success rate of 75% (range 0–100%)^20^, Brandes *et al.* of 66%.^29^ In contrast to these results Aldape *et al.* found prospectively a success rate of 91% in 995 patients with GBM.^35^

In the literature several reasons for the low success rate of MSP testing in paraffin-embedded tumour tissue of patients with GBM compared to fresh frozen tissue are discussed. Frequently only a very small amount of partially degraded DNA is recovered due to extensive necrosis and scarcity of malignant cells. Herrlinger *et al.* observed that 17% of the tumour specimens did not contain enough DNA.^33^ Especially in tumour biopsies tumour cells are not easily found.^24^ Hau *et al.* recommended a good quality paraffin embedded tissue that is not overfixed.^19^ The accumulation of normal cells in the tumour, including infiltrating lymphocytes, may complicate accurate assessment of MGMT.^28^ The best results with methylation-specific PCR are obtained with cryopreserved tumour specimens, thus avoiding the fixation-related deterioration of the quality of DNA.^36^ 8 of 14 our patients exhibited both the methylated and the unmethylated MGMT promoter. Our observation is in the same range as reports by Martinez *et al.,* Blanc *et al.,* Criniere *et al.,* Cankovic *et al.* and Gonzalez-Gomez *et al.* who found that the majority of the methylated tumours also exhibited an unmethylated band, which may arise from either normal cells within the tumour sample or from a tumour cell side population.^18^,^26^,^31^,^34^,^37^ Our experience shows that MGMT promoter methylation testing may be technically challenging. Several methods including multiplex ligation probe amplification MLPA, real time quantitative polymerase chain reaction (quantitative rt-PCR), have been proposed as potential alternatives to conventional MSP. These methods need to be critically evaluated in future studies and reliable cut-off values for the prognostication and the prediction have to be prospectively validated.^38^,^39^

Two of 33 (6%) patients of our study suffered from secondary GBM. This percentage was markedly lower than the incidence of 20% reported by Steinbach *et al..*^6^ In these two patients with secondary GBM in our study the MGMT promoter methylation determination was not feasible due to technical reasons.

The determination of the proliferative activity in form of MIB-1 evolved a low median score of 30.3 (range 12.1–49). This observation correlated well with the results reported by Ho *et al.*, demonstrating a cut-off value of ≥ 35 being related to worse outcome in unselected GBM patients.^40^ However, due to sampling differences, there has no clear prognostic impact of Ki-67 on the survival of GBM patients been detected.^10^,^22^,^41^

In addition to the MGMT methylation status, we compared clinical parameters of our long-term GBM patients like age, Karnofsky-performance score, ratio male/female, localisation of the primary tumour, extent of surgery, laterality of the primary tumour, incidence of cognitive impairment and of ischemic events, incidence of relapses, median survival, to those of other reports of long-term survivors with GBM. Most authors included patients surviving ≥ 3 years, Vertosick *et al.* those > 4 years, McLendon *et al.,* Steinbach *et al.* and Salvati *et al.* patients surviving ≥ 5 years and Morita *et al.* those ≥ 7 years.^6^,^8^,^11^–^13^

The median age at diagnosis of the patients of this series was 38 years, which is only slightly younger than the median of 41 years (range 37–51 years) observed in 10 different studies^5^,^6^,^8^,^10^–^13^,^18^,^23^,^42^ and 12 years younger than the average age at diagnosis of unselected GBM patients (median 53 years ± 0.55 years).^10^

One of the most important prognostic factors in cancer patients^43^, the Karnofsky performance score at the beginning of radiochemotherapy in our 33 patients reached median 90%. This equals the median of 90% (range 80–90%) observed in nine other studies of long-term surviving GBM patients^5^–^7^,^10^–^12^,^18^,^23^,^42^ and is clearly higher than the median of 76.1% of unselected GBM.^10^ The sex ratio male:female in our patient cohort was 67:33, quite similar to other studies. There was no predilection in laterality or in localization in a given cerebral lobe, as in the other series of long-term surviving GBM patients.^6^,^13^,^23^

The radiological parameter “extent of oedema > 1 cm” at diagnosis of GBM was present in 58% of our patients and did not impact on time to progression or survival in this series. In other studies of long-term surviving patients with GBM oedema was not investigated as a prognostic factor. However, in average patients with GBM, oedema larger than 1 cm has been reported to influence the survival, negatively.^44^,^45^

Of note, a gross total resection was achieved in 58% of the patients of this series. This has been reported accordingly in the series by Scott et al. with 40%, Salvati et al. with 46% and Hottinger et al. with 48% but not by Mc Lendon et al. with 27%.^8^,^10^,^12^,^42^ Obtaining a total gross resection appears to be of paramount importance for achieving a long-term survival in GBM patients. A considerably lower percentage of gross total resections of about 40% were recorded in studies of unselected patients.^3^ However, in this series four patients (12%) underwent only a biopsy of the primary tumour. In three of these four patients the MGMT status was not evaluable and in the remaining patient the MGMT promoter was unmethylated. This raises hope that even patients without tumour debulking and with unmethylated MGMT promoter status can eventually achieve a long-term survival.

61% of our patients relapsed locally. This was in line with three other reports of long-term surviving GBM patients specifying a percentage ranging from 45–73%.^10^,^12^,^42^ Of note, we did not observe distant relapses in the cohort of long-term surviving patients.

In 33% of our patients a cognitive impairment was recorded. A similar rate of 28% has been reported by Hottinger *et al.*^42^

Two of 33 (6%) of our patients suffered from an ischemic event, this was nearly identical to the 10% observed by Steinbach *et al.* but clearly lower than the 23% found by Hottinger *et al..*^6^,^42^ Further trials will have to evaluate the incidence of ischemic events in long-term surviving patients with GBM, to identify risk factors and establish preventive strategies.

In summary, this series of patients achieving a long-term survival after the diagnosis of GBM illustrates the validity of the prognostic factors developed in the nomogram by Gorlia *et al.*^15^ on the patients of EORTC and NCIC trials as well as of other series with long-term surviving patients with GBM: young age, extensive tumour resection, favourable performance status and treatment according to the standard of care, as well as a high percentage of glioblastomas with MGMT promoter methylation. The definitive role of MGMT promoter methylation in directing tailored chemotherapy in GBM patients will be elucidated in the large randomized international intergroup trial RTOG0525/EORTC26052 stratifying GBM patients by MGMT methylation status and randomizing for standard temozolomide in contrast to dose-dense temozolomide therapy. MGMT promoter methylation testing represents a substantial step forward in the treatment of patients with glioblastoma multiforme and enables us to better understand the mode of action of alkylating therapies and the course of the disease. Further, new treatment options exploiting the MGMT promoter methylation mechanism may add to the improvements achieved in this disease.

## Figures and Tables

**TABLE 1:** Patient characteristics

	**N (%)**
**Number of patients**	33
**Age (years) median (range)**	38 (22–66)
**Sex**	
Male	22 (66.7)
Female	11 (33.3)
**Performance status acc. to Karnofsky (%)**	
60	1 (3.1)
70	3 (9.1)
80	11 (33.3)
90	14 (42.4)
100	4 (12.1)
**Oedema**	
≤ 1 cm	11 (33.3)
> 1 cm	19 (57.6)
n.e.	3 (9.1)
**History of glioblastoma**	
Primary glioblastoma	31 (93.9)
Secondary glioblastoma	2 (6.1)
**Localisation of the tumour**	
Frontal	12 (36.4)
Parietal	4 (12.1)
Trigonal	2 (6.1)
Temporal	8 (24.2)
Occipital	1 (3.0)
Frontoparietal	1 (3.0)
Insula	1 (3.0)
Parietooccipital	2 (6.1)
Thalamus	2 (6.1)
**Side of tumour localisation**	
Right	10 (30.3)
Left	22 (66.7)
Bilateral	1 (3)
**Extent of resection**	
Biopsy	4 (12.1)
Subtotal	10 (30.3)
Total	19 (57.6)
**Cognitive impairment**	
Yes	11 (33.3)
No	22 (66.7)
**Stroke**	
Yes	2 (6.1)
No	31 (93.9)
**Initial chemotherapy**	
Temozolomide	6/33 (18.2%)
CCNU	15/33 (45.5%)
Fotemustine/Dacarbacine	12/33 (36.4%)

N = numbers, n.e. = not evaluable

**TABLE 2:** MGMT promoter determination

	**N (%)**
**Feasible**	14 (42)
Methylated	3 (21.4)
Unmethylated	3 (21.4)
Methylated and unmethylated	8 (57.2)
**Not feasible**	19 (58)
